# The importance of radiological controls of anastomoses after upper gastrointestinal tract surgery - a retrospective cohort study

**DOI:** 10.1186/1754-9493-4-17

**Published:** 2010-11-11

**Authors:** Joerg Doerfer, Thomas Meyer, Peter Klein, Nathaniel Melling, Alexander G Kerscher, Werner Hohenberger, Joerg OW Pelz

**Affiliations:** 1 Department of Surgery, University of Erlangen-Nuremberg, Germany

## Abstract

**Introduction:**

This study was designed to analyze whether routine radiological controls of anastomoses in the upper gastrointestinal tract an early detection of anastomotic leaks.

**Patients and Methods:**

135 patients who underwent upper gastrointestinal tract surgery were retrospectively analyzed. Patients in the first group (n = 55) underwent routine radiological control of the anastomoses. In the second group (n = 80) the radiological control was only performed in case of clinical symptoms or signs of anastomotic leaks.

**Results:**

The incidence of anastomotic leaks in the patients seen by us was 5.2%, equivalent to 7 of 135 patients In Group 1 leaks were seen in 4 of 55 patients (7,2%) in group 2 leaks were seen in 3 of 80 (3,8%). The radiological control of the anastomoses with contrast swallow showed the leakage in two cases. Twice the results were false negative. The sensitivity of computed tomography was 100%.

**Discussion:**

Routine radiological control of anastomoses with contrast swallow only has low sensitivity. This procedure should not be performed routinely any more.

The radiological control should be used in cases with signs of anastomotic leakage or with postoperatively impaired gastrointestinal passage.

## Introduction

Nowadays, resections and surgery involving opening of the lumen of the upper gastrointestinal tract are standardized procedures.

With continuous optimization of operating techniques the high complication rate and postoperative mortality was reduced. But anastomotic leakage still is one of the most feared postoperative complications [1-9].

By introducing mechanical staplers the initial technical problems of the operations were nearly completely eliminated [10,11]. Today, early detection of postoperative complications as well as their best possible treatment is the main issues.

The main procedure in the diagnosis of anastomotic leakage used to be the radiological control of anastomoses with water soluble contrast swallow [2,7,9,12-16]. Nowadays, two other reliable diagnostic tests (endoscopy and CT) are available [3,17-21].

A significant reduction of morbidity and mortality of anastomotic leaks necessitates not only exact knowledge of influencing factors but especially early detection and suitable therapeutically strategies [6,22-26]. For this reason many surgical departments perform routine radiological controls of anastomoses.

In their trial, Oestmann et al. could only detect 70% of all leakages in the first five days. One quarter of the leaks was only found due to repeated controls [7].

Other authors recommend a repetition of the procedure in patients with previously negative results and persisting clinical symptoms so as to eliminate the possibility of an insufficiently performed previous test [2,12].

This retrospective study was designed to determine whether routine radiological controls of anastomoses are efficient and whether there are more suitable diagnostic tests with a higher sensitivity and lower complication rate will be answered.

## Materials and methods

In this study the data of 135 patients were included over a period of two years. All patients underwent surgery of the upper gastrointestinal tract. In this period, there was no standard of evaluation of the anastomoses.

Patients that underwent elective surgery or emergency operations of the stomach, the duodenum or the pancreas involving opening of the lumen of the upper gastrointestinal tract with anastomoses to the esophagus and stomach were selected. Type of anastomoses was end-to-end, end-to-side, side-to-end, or side-to-side depending on preferences of the surgeon. No exclusions were made as to age, sex, comorbidities and dignity of the disease.

There were 73 male patients (54%) and 63 female patients (46%). The range of age was 32 to 91 years with a mean of 62.7 years.

The decision which operation was to be performed was made according to the kind of disease and to international standards. Of course the individual situation of the patients and intraoperative findings also played an important role.

We compared the patients from two wards. Patients from one ward underwent contrast swallow, patients from another ward had no swallow. There were no differences in treatments and a uniform distribution among the groups (Table 1).

**Table 1:** Table of all patients with different procedures (n = 135)

	Contrast swallow (N)	No swallow (N)
**Gastroenterostomy**	1	2

**Local Excision**	5	9

**Distal gastric resection**	21	29

**Gastrectomy**	12	16

**Duodenotomy**	2	4

**Duodenum preserving pankreatic head resection**	3	6

**Kausch-Whipple Procedure**	11	14

	55	80

The radiological control of the anastomoses with contrast swallow was performed on the fifth postoperative day into the corresponding group (n = 55). A dedicated upper gastrointestinal radiologist carried out all contrast examinations with the patient in multiple positions. Each swallow was performed initially with non-ionic water-soluble contrast.

In the other group (n = 80), no routine controls were planned. Radiological examination of the anastomoses was not performed on a routine basis, but only when leakage was suspected on clinical parameters. These parameters included tachycardia (heart rate > 100 beats per minute), fever (body temperature > 38°C), local or generalized peritoneal reaction during physical examination, leucocytosis (> 12 × 10^3^/ml), prolonged adynamic ileus (> 3 days postoperatively)

There were no restrictions made as to when or which test or in which way radiological controls, endoscopy, ultrasonography or CT scans were to be performed.

All medical records of the patients in whom radiological imaging of the anastomoses was performed were retrospectively reviewed. The presence or absence of anastomotic leakage was determined. Radiological anastomotic leakage was defined as radiological features suggestive for leakage in patients who did not develop clinical leakage. These radiological features were the presence of contrast outside the bowel lumen.

## Results

Two of the 135 patients died (1.5%). Fulminate cerebral bleeding occurred in one patient (54 years) after he underwent Billroth II resection of the stomach for a benign disease. Immediate neurosurgical treatment could not prevent a lethal outcome.

One 79 year old patient developed pancreatic fistula with abscesses and postoperative bleeding after duodenopancreatectomy for malignant disease. Development of pleural effusion as well as pulmonary and cardiac failure followed. The patient died albeit repeated laparotomies and drainage of the abscesses.

Five relaparotomies on other patients were performed due to postoperative bleeding, abscesses (twice, once due to anastomotic leakage), abscess with postoperative bleeding (due to anastomotic leakage) and wound infection with affection of gastrointestinal passage.

55 patients (40.7%) were in the group for primary radiological control. The contrast administration in these cases was routinely performed on the fifth postoperative day.

The other 80 patients (59.3%) were in the group without radiological controls. Five of these patients (3.7%) underwent a radiological control due to clinical signs of anastomotic leakage, meaning that a total of 60 patients underwent this procedure (44.4%). The overall incidence of anastomotic leaks was 5.2%, equivalent to 7 of 135 patients. In Group 1 leaks were seen in 4 of 55 patients (7,2%) in group 2 leaks were seen in 3 of 80 (3,8%).

Clinical signs and symptoms that may point to anastomotic leaks are shown in table 2. At the point of the radiological control eight patients clinically presented with signs of anastomotic leaks.

**Table 2:** Table of all patients with postoperative symptoms that may point to anastomotic leaks (n = 135)

	Number of patients	%
**No symptoms**	84	62,2

**Fever**	27	20

**Elevated white blood cell count**	38	28,1

**Chills**	4	3,0

**Sepsis**	4	3,0

**Change in drain secretion**	8	5,9

**Other**	9	6,7

Important signs showing anastomotic leakage were sepsis (n = 4) and a suspicious secretion of fluid in drains (n = 8).

No leakage was found in 57 of 60 controls. Twice leakage was proved by contrast medium outside the upper gastrointestinal tract. One control provided no conclusive results.

The results of the radiological controls were conclusive in all but one of the cases.

In 85.2% of the cases (n = 52) the contrast material flowed normally through the gastrointestinal tract. In seven cases (11.7%) changes like stenoses could be found. Postoperatively impaired gastrointestinal passage could be shown in 15 cases (25%), in 44 cases (73.3%) the contrast medium passed through the bowel in normal time.

In two controls contrast medium was seen outside the gastrointestinal tract (3.3%). The other 57 patients showed normal results in respect to leakage. None of the 59 cases with conclusive results showed signs of suture insufficiency.

After performing the radiological controls three specific complications were reported.

One aspiration of contrast material was seen, which developed without further complications. Mild pneumonia due to aspiration as well as hiccupping resistant to therapy was documented.

Pulmonary oedema or vomiting was not seen in our study.

Ultrasonography, computed tomography and endoscopy were used as further diagnostic tests. 13 patients underwent ultrasonographic controls. In two of these cases pathological findings were made that pointed towards an anastomotic leak.

Endoscopy was performed in 23 cases, which showed anastomotic leaks in three patients. 22 CT scans were performed and showed anastomotic leaks in 7 cases. All these alternative diagnostic tests had no complications.

Table 3 shows a summary of the sensitivity of the various diagnostic tests.

**Table 3:** Table of patients with detected anastomotic leaks (n = 7/135)

case	Radiological control	Ultrasound	Endoscopy	CT
**1**	detection	not performed	not performed	detection

**2**	detection	not performed	detection	detection

**3**	negative	detection	detection	detection

**4**	negative	not performed	negative	detection

**5**	not performed	detection	detection	detection

**6**	not performed	not performed	not performed	detection

**7**	not conclusive	not performed	negative	detection

The results show that computed tomography directly or indirectly detected the leakage in all the seven cases. The sensitivity of this method was 100% in both groups in our study.

Endoscopy showed the anastomotic leak in three out of five cases, which results in sensitivity of 60%.

The radiological control of the anastomoses with oral contrast material detected leakages in two cases two cases, both of which were false negative, as determined by CT scan. One control provided no conclusive results. Excluding this latter test, the sensitivity is 50%.

Two of the seven patients with anastomotic leaks underwent ultrasonographic controls. In both of these cases signs of anastomotic leakage were seen (sensitivity 100%).

In one of the leaks no further intervention was necessary. The leakage sealed without complications.

In another case the leak was treated endoscopically. Again another patient underwent CT-guided drainage of the abscess. Both leaks healed without operative intervention.

One patient had to undergo relaparotomy with an extended resection of the anastomoses.

Another patient was primarily treated endoscopically and with CT-guided drainage but the results was not satisfactory. Here too we performed relaparotomy.

## Discussion

A number of studies have raised concern about the limitations and potential dangers of routine contrast swallow examinations after oesophagogastric resection.

A recent systematic review of gastrointestinal anastomotic leaks confirmed that routine contrast radiology is still performed by most centres before the reintroduction of oral intake. It also revealed a lack of consensus on the type of contrast used or the timing of the examination, which varied from day 3 to the day 14 after operation [27].

The aim of our trial was to show whether radiological controls of anastomoses in the upper gastrointestinal tract performed on a routine basis are obsolete or still have an indication.

Literature research reveals many articles showing that radiological control of anastomoses is to be critically reviewed because of the low sensitivity and potential, test related, complications that may have lethal outcome [2,7,12-16].

It is also important to mention that the radiological controls should be done under standardized circumstances and that the results should be interpreted by an experienced radiologist with good knowledge of the operation and reconstruction techniques involved.

Due to postoperative changes such as swelling of the anastomoses or impaired gastrointestinal passage false negative results can occur [7].

Furthermore routine controls of anastomoses on the fifth postoperative day can not detect leakages that occur at a later point of time. In these cases it is advised to closely monitor the patient and initiate appropriate diagnostic tests only in cases with clinical signs of anastomotic leakage. If necessary negative tests must be repeated or other tests must be chosen [2,7,12].

The incidence of anastomotic leaks in the patients seen by us was 5.2%, equivalent to 7 of 135 patients.

In the literature, rates of 3-20% are found for anastomotic leakage after resections involving the upper gastrointestinal tract [3,7,11,12,16].

In our study 60 patients underwent a control of the anastomoses with contrast material. Eight cases were suspected to have a suture insufficiency at the point of the control.

Fever and elevated white blood cell count were the main parameters when it came to complications. These can also be elevated in the postoperative course without complications. A postoperative elevation of the C-reactive protein can persist till the third postoperative day [22]. After that it should reduce continuously.

In five out of the seven detected leaks a radiological control of the anastomoses was performed. One control was not conclusive due to bad imaging quality. The leak was found in two out of four cases resulting in a sensitivity of 50% in our trial. This matches the results from other trials [7,12].

A specific spectrum of complications for postoperative diagnostic tests with contrast material is reported on in literature [4,12,16].

In our group of patients that underwent this test (n = 60) three complications occurred. One aspiration of contrast material was seen, which developed without further complications. Mild pneumonia due to aspiration as well as hiccupping resistant to therapy was documented.

This relatively low complication rate (5%) is due to the careful testing, the good preparation of the patient and the individual choice of the most suited contrast medium by experienced radiologists.

Nowadays there are other diagnostic tests for controls of anastomoses apart from the classical method with oral contrast material, like ultrasound, endoscopy and CT [3,17-21].

Ultrasonography is a cheap and non-invasive diagnostic test. It is not routinely used in diagnostics for anastomotic leakage because postoperative changes often limit the imaging. Here, an experienced radiologist is necessary to obtain representative results.

In our trial ultrasound was used 13 times, twice in cases with anastomotic leaks. In both cases signs of leakage were seen. Because intervention is not possible and the imaging was limited as described above, additional computed tomography was used in both cases to optimize the diagnostic procedure.

By continuous optimization of technologies and teaching of physicians endoscopy has, in the last few years, evolved from a technically difficult method only used in specialized and hardly used for diagnosis of anastomotic leaks to a widespread and safe diagnostic test.

It is important to note that complications may occur during endoscopy too, especially perforation, bleeding and aspiration.

This method only allows an examination of intraluminal lesions. Conclusions on gastrointestinal passage are not possible. But the advantage lies in the possibility of interventional therapy as well as diagnostics. For example in a case of anastomotic leakage lavage of the leak and the surrounding cavity on a regular basis is possible as well as the application of fibrin glue.

We used endoscopic procedures in 23 cases. Five of the seven patients with leaks underwent this diagnostic test. In three cases (60%) the leak was detected. In two cases no leakage was found (40%). In one case the leak was treated with endoscopic lavage and application of fibrin glue till it sealed.

Nowadays, CT is widely spread and available in most German hospitals. It is a non-invasive method but application of contrast material has risks like renal failure, thyrotoxicosis and allergic reactions. Computed tomography is the most expensive of all described diagnostic tests. CT can not only the visualize leakage of contrast material but also complications of the leak and it allows imaging of thorax and abdomen in one examination. Another advantage is the possibility of placing a CT-guided drain.

CT was used 22 times in our trial including the seven patients with anastomotic leaks. In each of these cases the leak was detected either directly or indirectly, resulting in a sensitivity of 100% in our results. A CT-guided drain was placed in three out of seven patients. One leak was treated in this way till it sealed.

Routine radiological control of anastomoses seems obsolete when reviewing these results.

This diagnostic test should only be used in cases presenting with clinical signs of anastomotic leakage or in specific questions such as gastrointestinal passage time.

## Conclusions

Routine controls of anastomoses with oral contrast administration only have a low sensitivity. Based on these findings, we recommend that this test should not be used routinely any more.

This diagnostic test should only be used in cases presenting with clinical signs of anastomotic leakage or in impaired gastrointestinal passage.

Considering the various clinical presentations of the patients there are a row of alternative diagnostic tests available.

In our experience ultrasonography provides good orientation help in diagnosis of anastomotic leakage.

Endoscopy is an alternative diagnostic test which allows therapeutic intervention. Intrathoracic anastomoses are the main domain of endoscopy.

In our study CT proved to be the most apt diagnostic test especially in abdominal anastomoses. CT also provides the option of placing drainages.

Most importantly the decision on when a diagnostic test for anastomotic leakage is necessary and which one is the most suitable should always be made after careful clinical examination and strict evaluation of indication criteria.

## Competing interests

The authors declare that they have no competing interests.

## Authors' contributions

JD participated in the study design, data collection and analysis, and participated in drafting and revising the manuscript. TM participated in the design and coordination of the study. PK participated in the study design, data collection and analysis of the study. NM drafted the manuscript. AK participated in the study design, data collection and analysis, and participated in drafting and revising the manuscript. WH participated in the design of the study. JP conceived the study, was responsible for its design and coordination, participated in the analysis and interpretation of the data, as well as in drafting and revising all versions of the manuscript.

All authors read and approved the final manuscript.
